# Metabolomic and gut microbial biomarkers of smoking cessation treatment in long-term drug therapy: a study protocol for a randomized controlled trial

**DOI:** 10.3389/fpsyt.2026.1677235

**Published:** 2026-03-03

**Authors:** Johannes Peter, Alina Pritz, Michaela Hiebler, Mahdi Mahmoudi, Jürgen Fuchshuber, Sabrina Mörkl, Sandra Holasek, Elke Humer, Human-Friedrich Unterrainer

**Affiliations:** 1 Faculty of Psychotherapy Science, Sigmund Freud University, Vienna, Austria; 2 University Clinic for Psychiatry and Psychotherapeutic Medicine, Medical University Graz, Graz, Austria; 3 Addiction Research Hub (A-R-H), Grüner Kreis Ltd., Vienna, Austria; 4 Faculty of Medicine, Sigmund Freud University, Vienna, Austria; 5 Department of Psychoanalysis and Psychotherapy, Medical University Vienna, Vienna, Austria; 6 Comprehensive Center for Clinical Neurosciences and Mental Health, Medical University Vienna, Vienna, Austria; 7 Division of Medical Psychology, Psychosomatics and Psychotherapeutic Medicine, Medical University Graz, Graz, Austria; 8 Division of Immunology, Otto Loewi Research Center for Vascular Biology, Immunology and Inflammation, Medical University of Graz, Graz, Austria; 9 Department for Psychosomatic Medicine and Psychotherapy, University for Continuing Education Krems, Krems, Austria; 10 Department of Religious Studies, University of Vienna, Vienna, Austria

**Keywords:** addiction therapy, craving, gut microbiome, metabolomics, nicotine dependence, randomized controlled trial, self-efficacy, substance use disorders

## Abstract

**Theoretical framework:**

Cigarette smoking is the leading preventable cause of death worldwide, with nicotine dependence notably common among individuals with Substance Use Disorders (SUD). Smoking exacerbates both physical and mental health issues, further complicating the treatment of SUD. Current therapeutic approaches for SUD often prove inadequate, indicating a need for new strategies. Recent advancements in metabolomics and gut microbiome research have provided valuable insights into the biological mechanisms underlying addiction, warranting further investigation.

**Objectives:**

This study aims to investigate the therapeutic potential of smoking cessation for individuals with SUD, using a Cognitive-Behavioral Therapy (CBT) six-week group intervention within a therapeutic community. The research specifically explores the psychobehavioral, metabolic, and gut microbiome domains. It is hypothesized that smoking cessation will improve emotional regulation self-efficacy and reduce substance craving, mediated by changes in metabolic and microbiome profiles linked to brain systems of affect and reward.

**Methods:**

A randomized controlled trial (N = 100) will be conducted, examining outcomes such as clinical relapse rates as well as microbial and metabolic markers, investigating pathways of short-chain fatty acids, oxidative stress and inflammation, lipid, tryptophan, and one-carbon metabolism. Participants will undergo a CBT smoking cessation intervention, with pre- and post-assessments, compared to a control group receiving treatment as usual. Metabolomic and microbiome analyses will be conducted using blood and stool samples, alongside psychological assessments via questionnaires. Covariate analyses will be undertaken to control for metabolic and gut microbial effects of long-term psychiatric medications (antidepressants, mood stabilizers, antipsychotics, and opioid substitutions) present in the sample. Behavioral assessments will be conducted at a 3-month follow-up. The study is registered at clinicaltrials.gov under NCT06803706.

**Level of originality:**

This research will enhance our understanding of the complex interplay between smoking and mental health, offering potential for more effective treatment strategies for SUD. The current study’s focus on connections between metabolic and gut microbiome pathways with affect and reward is expected to yield valuable insights into addiction mechanisms and improve diagnostic and therapeutic practices.

**Clinical trial registration:**

## 1 Smoking cessation in substance use disorder: metabolome and microbiome mechanisms, therapeutic barriers, and effects on emotion and reward processing

### 1.1 Novel treatment approaches and improved outcomes in SUD are required

Cigarette smoking is the main preventable cause of death worldwide (1). Substance Use Disorder (SUD) is a psychiatric condition and goes along with dramatically reduced physical health and life expectancy (2), severe mental health problems (3), increased risk for suicide (4, 5) and high societal costs (6).

Besides apparent detrimental effects on physical health, smoking also contributes to reduced mental health and well-being (7), and it increases the propensity for the use of other substances (8). In line with this, nicotine dependence is highly prevalent in individuals with other SUD. Between 74% and 98% of those with SUD are also smokers (9, 10) and it is often observed that patients with SUD who overcome their primary addiction eventually die from a tobacco-related illness (11).

This situation also reflects the insufficiency of current treatment options in SUD, which are considered ineffective for too many individuals (12). With rising numbers of individuals diagnosed with SUD and societal costs worldwide over the past decades, the improvement of treatment outcomes and the management of SUD is therefore considered an important public global health priority (13).

### 1.2 Metabolic and gut microbial mechanisms in SUD

Investigating metabolic and gut microbiome mechanisms holds promise for advancing the field (14, 15). Metabolomics, as the investigation of the metabolites present in an organism, may provide a better understanding of psychiatric processes, including the mechanisms of craving and relapse in SUD (12, 16, 17). SUD, in particular, can be conceptualized as metabolic disorders, as exposure to virtually any addictive substance has an impact on the metabolism of the organism, ranging from central impact on organs such as the gastrointestinal system and the liver up to energy turnover on different (intra- and extracellular) levels, going along with effects on signalling and transmitter systems in the brain (14, 16).

The conceptualization of SUD as a metabolic disorder originates from innovative forms of management of narcotic addiction in the 1960s, when Dole and Nyswander found out that relapse can be overcome in many cases by compensation of the underlying metabolic aberrances through methadone maintenance, enabling a return to normal work and relationship functioning (14, 18). With ongoing advances in the field, recent studies were able to match metabolic profiles with clinical phenotypes and subtypes of opioid addiction, introducing potential for the development of new treatment and management strategies (19).

The consideration of gut microbial factors and their consequences for brain signalling and metabolism, also referred to as the microbiota-gut-brain axis, is a complementary perspective in this context (12, 15, 20). The insight that the microbiome shapes host metabolic, immune, endocrine, as well as central and autonomous nervous functions has led to a paradigmatic shift in neuroscience (21–25). Gut microbiota are integral agents in the metabolic processes of the human body. In detail, relevant classes of metabolites as well as the bacterial taxa involved in their production and proliferation, have been identified. Among others, gut bacteria-associated metabolites, such as short-chain fatty acids (SCFAs), polyunsaturated fatty acids (PUFAs), bile acids, and those involved in tryptophan metabolism, are highly relevant to psychiatric processes (20, 26). These metabolites affect energy turnover in the brain, as well as signalling and transmission, development, inflammation, and protection of brain tissue, as well as the permeability and integrity of the blood-brain barrier.

Metabolic and gut microbial mechanisms in addictive processes were identified in a broad range of relevant substances with regard to SUD, such as nicotine (27, 28), alcohol (29–31), amphetamines and psychostimulants (32, 33), cocaine (34, 35), and opioids (17, 19). Importantly, the current understanding of SUD and addiction in general is not limited to the biological dimension but – following the biopsychosocial model- takes into account psychological as well as social factors and interventions (36, 37). This enables meaningful research through the combined study of psychological interventions, as well as metabolome and microbiome perspectives and methods, as demonstrated by pioneering studies (38, 39).

### 1.3 Smoking cessation: therapeutic barriers and positive effects on mental health

While progress in the general understanding of SUD is necessary, smoking cessation interventions also offer a worthwhile way to improve outcomes in SUD treatment concerning physical health. Moreover, evidence suggests a therapeutic surplus value, as evidenced by improved mental health and favourable effects on reward processing (8, 10, 40). Nonetheless, smoking cessation interventions are not implemented widely and systematically in SUD treatment (41). Studies show that only modest percentages of SUD patients are reached by smoking cessation programs due to misconceptions of clinicians and organizational barriers such as limited training, lack of resources, and cultural norms that do not recognize smoking cessation as part of the therapeutic mission. Smoking in staff, therapeutic nihilism in mental healthcare workers, and subjective theories that smoking cessation concurrent with other alcohol or drug treatment may create a risk to sobriety were reported (41–43). The self-medication hypothesis, assuming that smoking can relieve stress and other symptoms related to poor mental health, is another influential belief (7).

However, empirical research shows a very different picture: tobacco smoking goes along with a repetitive vacillation of mood states (44). Smoking deteriorates mood and causes anxiety as well as stress and withdrawal symptoms rather than reducing them (45). As these feelings are reliably (yet temporarily) relieved by smoking, this creates the perception that smoking has psychological benefits, while, in fact, it is smoking that causes psychological disturbances in the first place. Thus, smokers seem to misattribute the ability of cigarettes to abolish nicotine withdrawal as a beneficial effect on mental health (40, 44). A growing body of evidence underlines this effect: starting to smoke increases mental illness parameters such as depressive and psychotic symptoms, anxiety and stress and is even a predictor of suicide attempts (46–48). Conversely, smoking cessation has been reported to improve a variety of mental health parameters in meta-analytic research, namely anxiety, depression, perceived stress, and positive affect. Concerning improved mood, as well as reduced anxiety and stress, the effect size of smoking cessation is comparable to the effect size of serotonin reuptake inhibitors and might thus, indeed, be therapeutic (7, 40).

### 1.4 Gateway effects of nicotine on brain reward circuitry

Effects of nicotine on the brain’s reward system have been studied extensively under the assumption that it might act as a ‘gateway drug’. The gateway hypothesis posits that certain substances lower the threshold for addiction to other, more severe psychoactive substances. The evidence supporting such effects of smoking is compelling (8, 49). It was found that nicotine increases dopamine release in the brain’s reward circuitry through functional alterations in the *nucleus accumbens* and the ventral tegmental area. This change enhances neural reward sensitivity, making individuals more susceptible to other drugs by promoting synaptic plasticity. The mechanisms involved herein include epigenetic modifications, such as histone acetylation, which influence the expression of certain genes, including the transcription factor ΔFosB, in reward neurons. Additionally, alterations in mesolimbic receptor systems, including nicotinic acetylcholine and AMPA receptors, as well as GABAergic inhibition, are also involved. Altogether, these molecular changes can lead to long-term structural and functional adaptations in neural networks, increasing the propensity for subsequently administered substances like cocaine (8). The relevance of these molecular mechanisms is underpinned by behavioural evidence from experimental animal research and epidemiological data. Levine and colleagues experimented with a combined gavage of nicotine and cocaine in mice and reported dramatically increased addiction-related behaviours such as conditioned place preference and locomotor sensitization (49). In human cocaine-naive users, it was found that regular smokers had a markedly higher probability of developing an addiction to cocaine. There is also evidence for increased reward and addiction to alcohol, psychostimulants, cannabis, and opioids through nicotine, and the maturing brain during adolescence seems to be especially vulnerable to these effects (50). Further support for gateway effects of nicotine derives from large-scale epidemiological data from annual US nationwide surveys. Here, it was found that smoking rates declined in parallel with non-medical drug use. It was reported that the decline resulted from the growing group of non-smokers, and their levels of non-medical drug use were at least four times lower than those of smokers (51).

While these mechanisms are well-documented, from the molecular to the epidemiological level, their findings seem to be hardly perceived in clinical contexts. Also, the role of nicotine in trajectories of SUD remains largely unclear, and it is currently unknown to what extent the effects of nicotine on reward processing are reversible. The reversibility of nicotine’s effects on reward processing is a critical question for this study, which hypothesizes that reversing gateway mechanisms may mitigate addictive factors and lead to improved outcomes in SUD treatment.

### 1.5 Associations between primary emotions, self-efficacy reward processing, and SUD treatment outcomes

It seems plausible but is currently speculative that the negative influence of nicotine on the brain’s reward systems might be reversed by smoking cessation. Whereas meta-analytic research clearly links smoking cessation to improved mental health (40), other studies suggest that this is due to alterations in emotional states, such as reduced emotional fluctuations and changes in the experience of positive and negative emotions over time (44, 52). Smoking affects nicotinergic and dopaminergic pathways in subcortical brain systems (45) which are involved in the biological foundations of primary emotions. Several findings point to associations between primary emotions, cigarette smoking, and addictive behaviour in general (53, 54). It is thus believed that primary emotions play an important role in SUD by influencing the regulation of stress and reward systems and have at least a mediating effect on substance craving and addictive behaviour (53).

However, the relationship between smoking cessation and primary emotions warrants further empirical investigation. Consideration of metabolic and gut microbial mechanisms may also bring further elucidation regarding this association.

### 1.6 Pilot study: Fuchshuber et al. (2024)

A recent pilot study from our research group showed significantly increased self-efficacy in individuals with SUD following a 6-week smoking cessation intervention during an inpatient stay within the setting of a therapeutic community, going along with reduced consumption of cigarettes per day (55).

**Figure 1:**
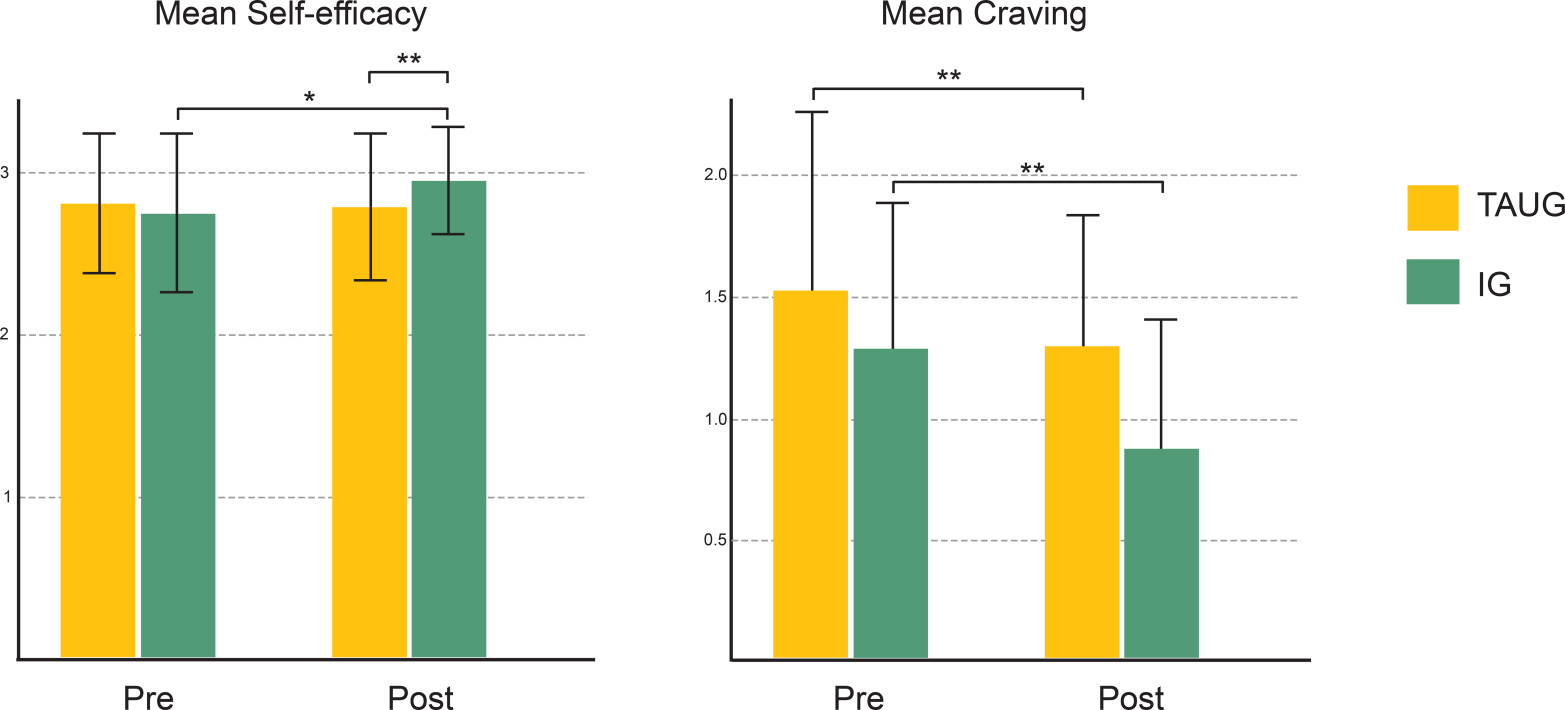
Results of the pilot study examining the effects of smoking cessation in SUD patients Left: Significantly interaction effect in self-efficacy (group x time); Right: Significantly decreased consumption of cigarettes per day; Decrease in general substance craving is more pronounced in the IG than in the TAUG by tendency (p = .18).

Longitudinal data from the tobacco cessation intervention (IG) vs treatment-as-usual control group (TAUG), group x time interaction effects showed significantly improved self-efficacy (2 x 2 ANOVA: *F*(1,53) = 5.86; *p* <.05; η_p_^2^ = .11) and decreased consumption of cigarettes per day (GEE: Wald-χ2 = 4.72, *df* = 1, *B* = 1.16, *p* <.05) in the intervention group (see **Figure 1**). While this pilot study had some limitations (pre-post design without randomization; no follow-up examination; lack of power), decreased general substance craving trends were also observable. This may be related to the aforementioned therapeutic effect of nicotine cessation, which could potentially reduce general addictive factors and improve SUD outcomes through biological and psychological mechanisms, including alterations in metabolic and gut microbial factors, brain reward processing, primary emotions, and perceptions of self-efficacy.

### 1.7 On the interactions between emotions, self-efficacy, reward processing, and addiction outcomes

Models of information processing in open, dynamic systems (56) might offer an integrative framework to explain the complex relationship between primary emotions, reward processing, coping, addictive behaviours, and self-efficacy. Such active inference frameworks typically rely on predictive information processing systems and the principle of minimizing free energy. It could be speculated that extensive negative emotional states can disrupt the brain’s ability to maintain stability through action and change, leading to maladaptive behaviours like substance use in order to artificially restore homeostasis. Psychoactive substances hijack reward circuits, thereby reinforcing behaviours that temporarily alleviate negative emotions but perpetuate long-term emotional instability. This is also in line with Baker and colleagues’ motivational theory of addictive behaviours, positing that ‘escape and avoidance of negative affect is the prepotent motive for drug use’ (57). Consequently, positive changes in primary emotions and reward processing following smoking cessation should lead to improved substance use outcomes. In line with this, meta-analytic research found that substance use outcomes improved in two of the nineteen trials and remained unchanged in seventeen (10). Changes in primary emotions and increased emotional stability, in combination with the experience of positive change through personal agency and undergoing successful smoking cessation, might also increase self-efficacy —the belief in one’s ability to influence outcomes. It might recalibrate an individual’s predictive models of emotional states, as described in active inference frameworks (56). Consequently, we hypothesize that this recalibration fosters greater self-efficacy by reducing reliance on maladaptive coping strategies, enabling individuals to perceive themselves as agents of change rather than victims of their emotions. By altering the brain’s allostatic predictions, individuals gain a sense of control, which is pivotal in overcoming the cycles of craving and relapse characteristic of SUD.

### 1.8 Neural, metabolic, and gut-microbiome-mediated pathways of nicotine on affect and reward

#### 1.8.1 Mesolimbic nicotinic receptor systems

There is evidence that the direct actions at nicotinic acetylcoline receptors (nAchRs) of nicotine and its metabolites can increase negative affectivity, downshift hedonic set points, and increase sensitivity to social defeat stress (58). The neuroadaptions in nicotinic pathways in the brain caused by chronic smoking are associated with occurrence of depressed mood, agitation, and anxiety shortly after a cigarette is smoked (40). Experimental studies suggest a sensitization to negative emotional cues during nicotine withdrawal (52, 59). Functional alterations of the reward system are also supported by brain imaging studies (60). The fluctuations in the psychological state of smokers are known as the withdrawal cycle. The upregulations of nAchRs, and withdrawal symptoms were found to return to normal levels by three weeks after smoking cessation (61, 62).

Negative emotions, particularly in the absence of emotion regulation strategies, are often preceding substance misuse (53). The role of negative affects is also stressed by an influential addiction theory, which posits that escape and avoidance is the prepotent motive for drug use (57).

#### 1.8.2 Oxidative stress, lipid and inflammatory pathways

A multi-omics study compared metabolite levels between cigarette smokers, e-vapers, former smokers and never smokers (27). Metabolic concentrations showed distinct smoking dose-dependent distributions of elevated inflammation markers and pathways. In smokers, apart from nicotine and its downstream metabolites, metabolites derived from toxicants (e.g. p-cresol sulfate), increased metabolites of oxidative stress and detoxification pathways (pathways of gluthatione conjugation) were found. Lower caroten-diol levels indicated oxidative depletion of antioxidants. Oxidative stress responses are known to be associated with exacerbation of withdrawal symptoms and enhance reward seeking (63). Lipidomics analyses revealed increased concentrations in oxidized derivatives of polyunsaturated fatty acids (PUFAs). More precisely, eicosanoid derivates such as 14,15DHET or 9-HODE, known to be involved in inflammatory signaling, were elevated. Circulating CRP and haptoglobin were also increased, reflecting chronic inflammation in smokers. Strongly elevated levels of N-acetylputrescine were also observed, a metabolite related to polyamine metabolism and influencing cell proliferation, immune function, and stress responses (27). Similar and confirmatory patterns of increased inflammatory activation with elevated CRP and interleukin-6, and lipid profiles (cholesterol, triglycerides, LDL) in smokers vs. controls were recently published (64).

Activation of oxidative and cell stress, and lipid inflammatory pathways are linked in complex ways to reward circuitry. There is evidence for associations with blunted reward and increased propensity for immediate gratification via dopamine pathways in the nucleus accumbens and medial prefrontal cortex, but also increased negative affectivity, learned helplessness, and proneness for depression (65, 66).

#### 1.8.3 Gut microbiome pathways: SCFA, tryptophan, and bile acids

Few small studies have directly examined the effect of smoking cessation on the microbiome (67, 68), but there is a growing body of evidence for general smoking-associated gut microbial alterations (69). A large mendelian randomization study provided mechanistic insights for a cross-talk and causal relationship between bacterial species and smoking behavior. Fan et al. (28) reported that lower abundances of *Actinobacteria* may cause increased smoking, with a potential positive feedback effect (further lowered abundances of *Actinobacteria* by smoking). Bacterial species and also belonging metabolic pathways were identified: *Actinobacteria* and *Peptococcus* were associated with alterations in tryptophan and tyrosine metabolites, and *Peptococcus* with effects on valerate (a short-chain fatty acid SCFA). The authors concluded, that these bacteria, via the identified metabolites, play a causal role in the action pathway from the gut microbiome to smoking behavior (28).

Tryptophan metabolism, and the downstream kynurenine pathway is a key metabolic pathway in the broader addiction literature (70). It is known that drugs of abuse dysregulate tryptophan metabolism and kynurenine metabolite concentrations via oxidative stress and immune responses, with immediate effects on the dopamine reward system. A recent study provided additional evidence for disturbances of tryptophan and kynurenine metabolism through smoking (71). Disrupted kynurenine pathway metabolism was also found in depression (72), altogether pointing to shared pathways of affect and reward in addiction and other psychiatric conditions.

Short-chain fatty acids (SCFA) are produced by the bacterial fermentation of dietary fiber, and are known as key gut-brain signaling mediators with a high relevance for reward and addiction (73–75). SCFA are lowered in smokers via gut microbial effects (76). They have anti-inflammatory properties, protect against oxidative stress and modulate transcriptional regulation in the brain. SCFAs exert epigenetic effects, stimulating memory and synaptic plasticity by inhibiting histone deacetylases (HDAC) or modulating DNA methylation, thereby altering the expression of addiction- related genes (*e.g.*, CREB transcription factor, c-Fos, dopamine receptors and μ-opioid receptors) and persistently reshaping reward circuit plasticity (77).

Recent studies point to elevated cholesterol and disruptions in bile metabolism (associated with microbial alterations) induced by smoking (64, 78). Bile acids are known agents of gut-brain signaling, and disruptions in bile acid profiles were found in humans with anxiety and depression (79, 80).

#### 1.8.4 One-carbon metabolism: methylation as a mediator of reward

One-carbon metabolism, with primary methyl donors such as choline and folate, is also implicated in the biology of SUD (14). These compounds are vital for epigenetic regulation of learning mechanisms, and have been shown to impact responses to substances of abuse (81, 82). Smoking goes along with lower choline levels, higher homocysteine, and generally altered one-carbon metabolism (83, 84). Nicotine can indirectly influence cellular methyl group metabolism by altering the expression and activity of DNA methylation machinery, which affects the utilization of methyl donors like S-adenosylmethionine (SAMe). For example, nicotine exposure down-regulates DNA methyltransferase (DNMT) gene expression and reduces global DNA methylation in human cells, a process that may reflect changes in methyl group availability or DNMT demand for SAMe-dependent methylation reactions (85). Decreased methylation capacity is implicated in reward and stress pathways (14, 86). Choline is also the precursor of Acetylcholine, a neurotransmitter central to emotion, attention, and reward. Low choline and Acetylcholine are linked to anxiety, inflammation-related mood dysregulation, and altered dopaminergic reward signaling (87, 88).

The gut microbiome is an important player with regard to choline metabolism, as choline is consumed by gut microbes to produce TMA, which limits host choline bioavailability. The amount of microbial TMA production depends on microbiota composition, influencing choline and related one-carbon metabolites in the host (89, 90).

## 2 Research aims

**Figure 2:**
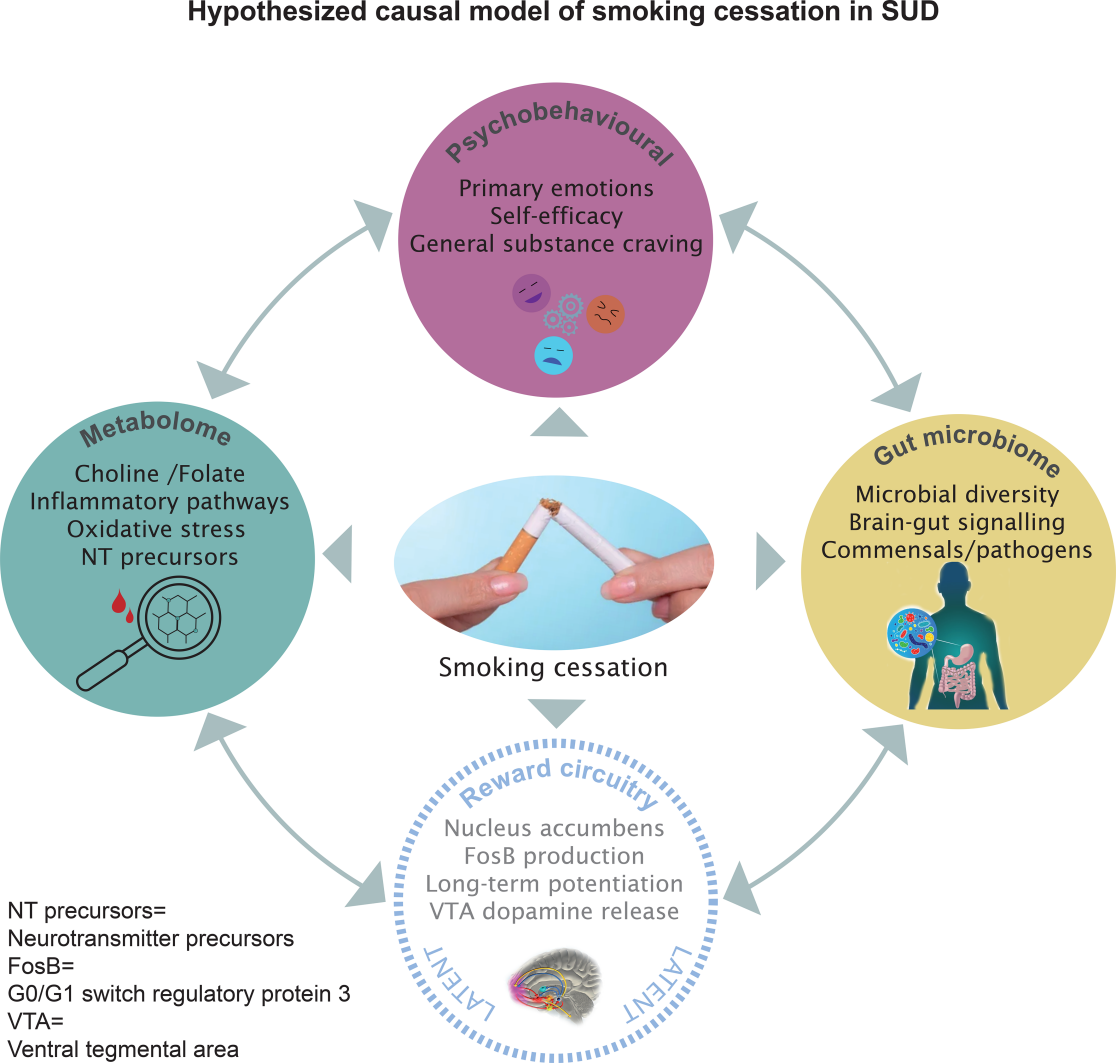
Hypothesized causal relations and interconnected mechanisms related to smoking cessation and SUD on metabolic, gut microbial, and psychobehavioural levels. Brain reward circuitry (‘gateway’) effects are also hypothesized but only indirectly observable (latent).

The present study attempts to clarify the mechanisms of action and therapeutic outcomes in SUD patients undergoing a 6-week behavioral smoking cessation program during their stay within the setting of a drug-free therapeutic community. The study aims to examine the mechanisms of action and therapeutic outcomes in three domains: the psychobehavioural domain, the metabolic domain, and the gut microbial domain. The existence of an additional fourth latent domain, namely reward processing, is assumed. As the causal interconnections between the domains and single variables are complex and innovative (see **Figure 2**), the investigations will be driven by clear hypotheses but will as well be largely of exploratory character.

### 2.1 Study objectives

#### 2.1.1 Psychobehavioural domain

In line with previous empirical findings from our pilot study and the research literature, we hypothesize changes in primary emotions, self-efficacy, and general substance craving (7, 10, 55). More precisely, we assume increases in positive emotions and decreases in negative emotions, greater emotional stability, and reduced psychiatric symptom burden post-intervention. Reversed gateway effects of nicotine on reward processing may contribute to these results due to shifts in neurotransmitter systems and mesolimbic and mesocortical pathways (8, 45). Based on empirical observations and theoretical frameworks of inference of allostasis, we further hypothesize that enhanced self-efficacy facilitates sustained behaviour change, reducing reliance on maladaptive coping strategies, resulting in reduced general substance craving, nicotine addiction, and reduced clinical relapse rates after smoking cessation. The secondary aim of the study involves replicating and extending the findings from our previous study (55). This includes the examination of the clinical long-term outcome and success of the smoking cessation intervention as reflected in group differences at 3-month follow-up.

#### 2.1.2 Metabolic domain

The notion of addiction as a metabolic disorder requires further investigation in human subjects and in the clinical field, which represents an emphasis of investigation in this study (14, 16). Analyses will be performed with regard to metabolic profiles in SUD patients during their inpatient-stay in a drug-free therapeutic community and the additional contrasting between-group effects of smoking cessation. While the examination of metabolic factors is largely exploratory, particular attention will be paid to metabolic markers and cluster analyses corresponding to psychiatric symptoms, as well as to pathways of choline and folate metabolism. Metabolic correlates of primary emotions and psychiatric symptoms will be identified in exploratory analyses. In line with previous research, we hypothesize that smoking cessation goes along with increased choline levels and altered choline/folate metabolism (14). Downstream analyses will be focused on metabolites of immune and inflammatory regulatory pathways, oxidative stress, neurotransmitter precursors and metabolites and enzymes (tryptophan, kynurenic and quinolinic acid, indoleamine-2,3-dioxygenase, and tryptophan-2,3-dioxygenase).

#### 2.1.3 Gut microbial domain

In line with previous studies, we hypothesize that smoking contributes to gut microbial imbalances, reduced general bacterial diversity and abundance of commensals, and growth of pathogens (91). We hypothesize that reversed effects in these imbalances will be observable after smoking cessation. Further emphasis will be put on the identification of microbial markers corresponding with general substance craving, history of smoking and success of smoking cessation. Gut microbial correlates of primary emotions and psychiatric symptoms will be identified in exploratory analyses. Analyses will further focus on bacterial genera with a known role (as derived from literature) in the metabolism of SCFA, PUFA, bile acids, and tryptophan (metabolites) (20). Combined metabolome-microbiome analyses will ascertain associations between bacterial abundances and concentrations of relevant metabolites.

## 3 Originality and scientific innovation

The pilot study by our group (55) showed that a smoking cessation intervention during addiction rehabilitation is associated with a significantly increased perception of self-efficacy and leads to a reduction in the number of cigarettes smoked per day. While much of the literature to date is still derived from animal studies with limited power (16), a new approach to metabolomic studies in humans suffering from SUD is expected to bring advances in the understanding of addiction mechanisms, as well as in diagnostics and treatment. With the proposed steps outlined herein, we are entering new fields in the area of addiction, as well as investigating the microbiome, and contributing to the research in the emerging psychiatric sub-discipline of *Metabolic Psychiatry* (92, 93), which explores the overlap between mental health issues and metabolic dysfunctions. Given the complexity of this research area, this approach could provide valuable insights into the biochemical processes and metabolic changes associated with SUD. Pioneer’s work demonstrates how the metabolomic perspective can lead to a multi-level understanding of psychiatric processes by integrating empirical evidence regarding the relative presence of metabolic molecules in the body, which have relevance and known causal role in central nervous metabolic and signalling processes (as identifiable through metabolic downstream/pathway analyses via bioinformatic databases) with synchronously collected behavioural data from variables such as response to therapy and psychological factors (38, 94).

The empirical investigation of the influential gateway hypothesis (8, 49) also plays a crucial role and by analyzing how the use of one substance influences the transition to other addictive substances, new strategies for prevention and treatment can be developed. Therefore, the extension of the pilot study by the analysis of the metabolomic and gut microbial biomarkers promises to deliver important insights into SUDs, which can be applied to treatment and preventive options.

The expected findings will also provide new foundations for the implementation of smoking cessation in the fields of addiction management and psychiatry and thus contribute to the reduction of therapeutic nihilism and good decision-making in healthcare policy.

### 3.1 Rationale for selection of this cohort

The undertaking to study metabolic and gut microbiome factors in SUD patients undergoing smoking cessation is associated with several methodological difficulties. SUD patients constitute a group that is distinct from other populations, characterized by psychiatric comorbidity, history of chronic substance exposure, altered metabolic and inflammatory regulation, and frequent polypharmacy. It must be expected that these factors are the source of significant biological variance, and previous studies that have examined metabolic or gut microbiome factors of cigarette smoking, and cessation, have circumvented this issue by studying non-SUD patients (27, 64, 67). On the other hand, individuals with substance use disorders (SUD) exhibit very high rates of tobacco use, nicotine dependence, and relapse (95), yet the biological mechanisms underlying smoking cessation in this population remain largely unexplored. Moreover, smoking cessation as an add-on therapy holds promise for a surplus value for SUD outcomes and trajectories through a hypothesized reversed *gateway* effect of nicotine cessation.

To date, the research literature lacks interventional RCTs on behavioral smoking cessation interventions including metabolomics and microbiome data in a longitudinal design. If metabolic or metabolomic markers are to inform clinically useful biomarkers or interventions, they must be validated under complex and real-world conditions.

### 3.2 Innovative aspects of the therapeutic community approach for SUD

The immanent assumption of the therapeutic community approach (36) is that SUD reflects deeper disturbances in identity and personality structure, values, emotional regulation, and social functioning rather than being only a biomedical dependency. Therefore, the central mechanism of change is “community as method,” meaning that recovery occurs primarily through structured social living, mutual self-help, and social learning processes in which peers provide feedback, role modeling, accountability, and reinforcement of pro-social behavior. The practical approach in a SUD recovery institution – which represents the actual environment for participants of the present study – goes along with structured leisure, work and community activities, as well as meals and exercise. This structure might contribute to social learning and establishing daily routines, a sense of goal-directedness and motivation for sustained drug abstinence (36). But it is also in harmony with metabolic and nutritional psychiatry (93), and microbiome perspectives, as it can be hypothesized that this structured way of life supports the restoration of circadian rhythms, metabolic and gut microbial homeostasis, and normalization of reward circuitry (96, 97).

## 4 Methods and research design

### 4.1 Type and design of the study

**Figure 3:**
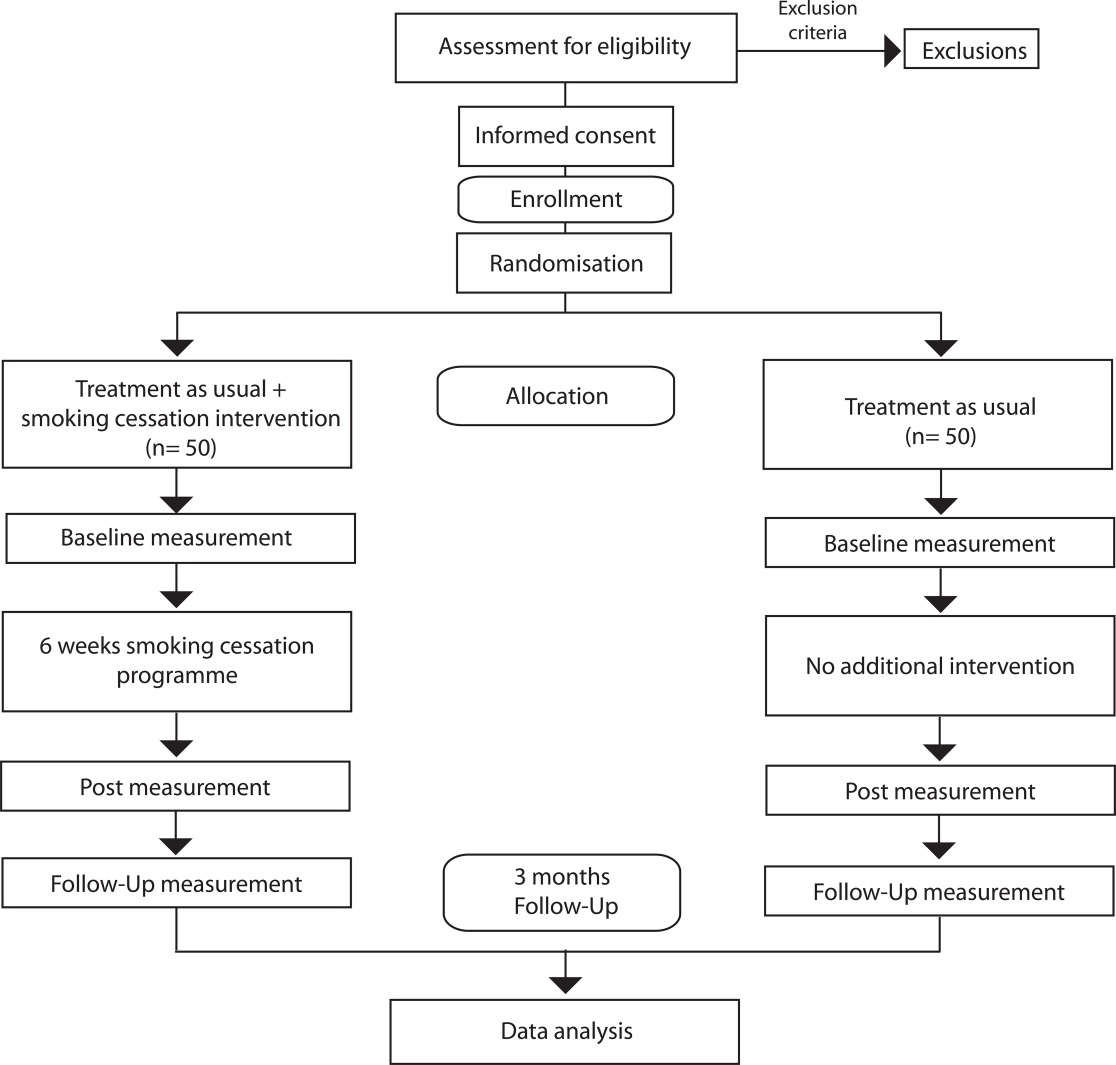
Flow diagram of the trial.

The present study is a randomized controlled experiment in N = 100 SUD- inpatients during a therapeutic community treatment with one intervention group that will receive an additional 6-week smoking cessation intervention and one control group that will receive treatment as usual. The study design consists of a 2 x 2 mixed subject design with repeated measures for the biological analyses and a 2 x 3 mixed subject design with repeated measures for the psychobehavioural analyses (see **Table 1**). In both groups, an examination of metabolic and gut microbial profiles, as well as psychological variables, will take place. Biological parameters will be ascertained from blood and stool specimens for nuclear magnetic resonance (NMR) spectroscopy metabolomic analyses and from stool specimens for 16s-mRNA (hypervariable V3-V4 regions) sequencing and subsequent gut microbial profiling. Psychobehavioural and biological data will be collected pre & post smoking cessation, and additionally, there will be a follow-up measurement of the psychobehavioural variables 3 months upon completion of the study (see **Figure 3**: Consort Flow Chart for further illustration).

**Table 1:** Overview of the statistical design.

Study group	Pre- intervention baseline	Post- intervention after 6 weeks	Follow- up after 3 months
Intervention Group (N = 50)	Psychobehavioural Data	Psychobehavioural Data	Psychobehavioural Data
	Metabolic/Microbial Data	Metabolic/Microbial Data	
Control Group (N = 50)	Psychobehavioural Data	Psychobehavioural Data	Psychobehavioural Data
	Metabolic/Microbial Data	Metabolic/Microbial Data	

green cells: biological data; light brown cells: self-report data.

### 4.2 Recruitment

The recruitment and examination will involve patients from the association “Grüner Kreis”, who are housed at the Johnsdorf facility. All patients are diagnosed with a form of SUD (F1x.x) by a licensed psychiatrist according to the International Classification of Diseases version 10 (98). Furthermore, participants have to explicitly state that they want to quit smoking. After administering the psychological questionnaires and collecting demographic data, patients will be randomly assigned to either the intervention or control group. The allocation to the groups will be carried out using computer-assisted block randomization (https://www.randomizer.org). The location for the examinations (blood and stool samples) is the Otto Loewi Research Center at the Medical University of Graz. The transfer of participants (Johnsdorf facility – Otto Loewi Research Center/Med Uni Graz) will be carried out by the association’s own buses from Grüner Kreis. Upon completion of data collection, the intervention group will undergo a 6-week smoking cessation program, while the control group will only receive treatment as usual. After the intervention phase, participants will again complete psychological questionnaires, and blood and stool samples will be collected at the Otto Loewi Research Center. Three months later, a follow-up assessment of the psychobehavioural variables will be conducted.

### 4.3 Intervention

During the inpatient stay at the ‘Grüner Kreis’ community, the patients will be allocated to an intervention group and a control group. Patients of the intervention group will take part in an additional 6-week behavioural therapy program for smoking cessation. Patients of the control group will undergo treatment as usual. The standard treatment within this therapeutic community—which all participants will receive—consists of group therapy (once a week), individual psychotherapy (once a week), counseling by social workers, psychiatric consultations, as well as sport-, art- and work therapy. For further information on the principles of therapeutic community-based addiction treatment, see, e.g., DeLeon & Unterrainer (36).

The “smoke free in 6 weeks” program is a standardized behavioural therapy intervention (see Batra & Buchkremer (99) for detailed instructions) that takes place once a week for 1.5 hours over 6 weeks and is provided by the Austrian health insurance provider *Österreichische Gesundheitskasse* (ÖGK). Held in the therapeutic community, it will employ principles of cognitive-behavioural therapy (CBT) coupled with personalized recommendations for nicotine replacement. The Austrian health insurance standard for therapy will be guiding the program’s content, which is tailored for inpatient clients. Specifically, the following interventions based on CBT techniques will be applied in the weekly group sessions consisting of 10 to 15 participants: Psychoeducation including information on tobacco addiction, health risks, advice on relapse prevention and the handling of craving, motivation building, behavioural observation, setting a personal quit-smoking day, behaviour modification through the development of alternative actions, training of progressive muscle relaxation, strengthening of self-efficacy and information on the topics of physical activity and nutrition. Finally, to increase intrinsic motivation, each session will include an analysis using a calibrated carbon monoxide measurement. The primary aim is to help clients achieve smoking cessation, favouring this approach over gradual reduction methods. To support ongoing abstinence and motivation, participants will be directed to additional resources such as the Smoke-Free App, Smoke-Free Phone, and regional outpatient cessation services. This program will be led by a clinical psychologist who is specifically trained in conducting the program.

### 4.4 Data collection

#### 4.4.1 Psychobehavioural variables – psychometric measures

In addition to anamnestic data, the following previously validated instruments showing good psychometric properties will be applied:

Self-efficacy: The Self-Efficacy Scale (SWE (100)) is a self-report instrument for measuring general optimistic self-confidence. It consists of 10 items and measures optimistic competence expectancy, i.e., confidence in solving difficult situations with one’s own abilities.

General substance related craving: The MaCS (Mannheim Craving Scale (101)) is an instrument for measuring substance related craving. It consists of 12 items and four additional items and is rated on a 5-point Likert scale.

Tobacco dependence: The Fagerström Test for Cigarette Dependence (formerly Fagerström Test for Nicotine Dependence; FTND (102)) is a self-assessment instrument measuring tobacco dependence intensity. It consists of 6 categorically scaled items that assess the number of smoked cigarettes per day (CPD), compulsive use, and dependence intensity.

Psychiatric Symptoms: For the evaluation of psychopathology, the ICD-10 Symptom Rating (ISR (103)) questionnaire was utilized. In accordance with the ICD-10, this self-assessment questionnaire, comprising 30 items, is designed to comprehensively assess the status and severity of mental disturbances. Symptoms of the six symptomatic patterns – depressive, anxiety, obsessive-compulsive, eating disorders, somatoform syndrome, and post-traumatic stress disorder – are assessed.

Primary Affects: The German version of the Brief Affective Neuroscience Personality Scales including a LUST Scale (BANPS–GL (104)) is a self-report questionnaire to measure all seven primary affects proposed by Jaak Panksepp (105) as personality traits.

Smoking behaviour: As previously in the pilot study, participants will be asked regarding their daily smoked cigarettes in the last two weeks (interval scaled). A second, dichotomous single-item question will assess if participants have been completely abstinent from tobacco smoking in the last two weeks.

#### 4.4.2 Dietary assessments

Dietary assessments will be performed using the Vienna Food Record (106) a food frequency questionnaire which takes Austrian-specific eating habits into account. Participants will be asked to document their food intake during the week prior to the assessments of blood and stool.

#### 4.4.3 Collection of biological samples

In order to collect the blood and stool samples, participants will be instructed to fast for at least 12 hours prior to venipuncture, and the blood samples will be drawn in the morning.

A maximum of 70 ml, equivalent to approximately 5 tablespoons of blood, will be collected into EDTA-coated tubes. Blood samples will be centrifuged at 4 °C for 15 minutes at 4000 rpm within 30 minutes of phlebotomy, and plasma aliquots will be immediately frozen at −80 °C until analysis.

Furthermore, approximately 1 g of stool will be collected from each participant in with stool sample kits with DNA/RNA stabilization buffer ensuring stabilization of microbial DNA at ambient temperature (OMNIgene^®^GUT, DNA Genotek Inc., Canada), immediately frozen and stored at -80 °C until further processing. For information regarding the further processing and downstream analyses of the biological data, see 4.7.5 and 4.7.6.

### 4.5 Inclusion and exclusion criteria

*Inclusion Criteria:* Participants must provide informed consent. They must have a diagnosis of a form of substance use disorder (F1x.x) by a licensed psychiatrist according to ICD-10 (98). Participants must be at least 18 years old, can be of either sex, and must have sufficient knowledge of the German language. All participants must explicitly express their willingness to quit smoking. A minimum duration of abstinence from the primary substance and stable dosage of long-term medications for at least 3 months prior to inclusion is mandatory.

*Exclusion Criteria* include lack of consent, age below 18, or inability to provide informed consent. Participants with acute psychotic symptoms or acute suicidal tendencies are also excluded. Further exclusions are cardiovascular disease, pregnancy or breastfeeding, severe mental or organic illnesses (such as epilepsy, brain tumours, recent major surgery), tumour diseases, dementia (Mini Mental Score <20), severe autoimmune diseases or immunosuppression, acute infections, or acute diarrhea. Individuals with prior gastrointestinal surgery (except appendectomy), probiotic intake within the last 6 months, or those consuming dietary supplements, probiotics, antibiotics, or prebiotic supplements during the study will also be excluded.

### 4.6 Risk assessment

Providing a stool sample carries no risk. However, blood sampling can result in a bruise (hematoma), and depending on the condition of the veins, it can be more or less painful. This will be performed by an experienced study physician in accordance with WHO guidelines (107), who will also offer immediate support if necessary. Completing online-questionnaires can lead to fatigue and tiredness. However, participants have the option to take breaks or to interrupt or discontinue completing the questionnaires at any time. Therefore, the risk-benefit ratio is considered positive, and the risk of the study is considered as low for participants.

### 4.7 Statistical analyses

#### 4.7.1 Power calculation

As the approach of this study is biopsychological, we drew on selected data from different domains from relevant studies to estimate effect sizes: metabolic differences between active smokers and former smokers, changes in gut microbiome parameters, and a psychological variable during smoking cessation. A recent multi-omics study (27) compared metabolite levels between cigarette smokers, e-vapers, and former smokers. N-acetylputrescine metabolite concentrations showed a clear association with smoking-related metabolic variation. Because raw metabolite concentrations were not given, effect sizes of standardized differences between cigarette smokers and former smokers were estimated from the correlation between N-acetylputrescine and the combustion-related principal component (PC1), giving an estimated approximate standardized mean difference of Cohen’s d ≈ 0.73, indicating a medium-to-large effect size. This suggests substantially higher N-acetylputrescine levels in cigarette smokers compared with former smokers, consistent with persistent alterations in polyamine metabolism in active smoking.

For microbiome changes after smoking cessation, Sublette et al. (67) reported increased abundances of Bacteroidetes in successful abstainers (n=14 of 26 at week 12). The increase in relative abundance of Bacteroidetes from baseline to 12 weeks in smokers who quit compared with those who continued smoking corresponded to an approximate standardized effect size of r ≈ 0.41 (based on regression estimate β = 7.4 percentage points, SE = 3.5), suggesting a moderate effect.

Psychological self-efficacy increased during smoking cessation in a pilot study by our own research group (55) and showed a medium-to-strong effect (η_p_^2^ = .11; converted to Cohen’s d ≈.70).

A-priori sample size calculations were performed with G-Power (108) (version 3.1.9.6), for a mixed-design ANOVA with one interaction between group (2 groups) and assessments (2 repeated measurements). Considering the effects mentioned above, the mixed-design ANOVA was set rather conservatively for a medium effect size (Cohen’s F = 0.25), a statistical Power (1-β) of 0.9, a α of 0.05, an intra-correlation coefficient of 0.5 and nonsphericity correction of 1, resulting in a minimum sample size of 46 subjects per group, and a critical F = 4.06. Adjusting for expected attrition of about 10%, a total sample size of size of 100 is set. The many (mostly biological) variables measured in this study will expectably require statistical corrections; this will also be compensated by statistical methods exceeding classical linear approaches, namely machine learning feature detection and modelling methods (109).

#### 4.7.2 Diet

Nutritional variables will be a highly relevant source of variance in the study, particularly for metabolome and microbiome parameters. Food compounds, energy and nutrient intake will therefore be analysed from food frequency data by the nutritional software nut.s ^®^v1.32.95 (110). Nutrient intake will be adjusted for total energy intake, and nutrients with excessive missings will be excluded.

It appears inadequate to consider dietal factors merely as control variables, as nicotine is known to interact strongly with regulation of appetite and satiety, and smoking cessation usually goes along with a weight-gain (111). Nutritional variables are therefore candidate mediator or explanatory variables, and it is important to quantify how much of the group effects will be explained by energy intake and nutrients. For this purpose, the variance explained by nutrition will be quantified via Partial distance-based redundancy analysis (db-RDA) and/or PERMANOVA (for both microbial beta diversity and metabolite distances). Variance will be decomposed by stepwise separation of variance explained by group, variance explained by nutritional variables (energy, nutrients, selected compounds), control variables (see below), and shared variance.

Additional adjustment analyses will be performed in candidate biomarkers of smoking cessation group effects emerging from metabolome and microbiome analyses (at the level of metabolites, or specific bacterial taxa). Linear mixed effects models (lme4 package in R (112)) will be iteratively tested (total effect vs. nutrition-adjusted models) for shrinked coefficients of group effects for this purpose.

#### 4.7.3 Controlling for potentially confounding variables, and multiple testing

Proper analysis of the research questions in the SUD sample will be hampered by several sources of variance, such as diet, psychiatric diagnoses, medications, and history of substance misuse. These variables will be held constant as control variables.

Psychiatric comorbidity is highly present in the population. Psychiatric symptom burden and classes of psychiatric diagnoses are associated with systematic metabolic and microbiome profiles (72, 113, 114). Classes of psychiatric secondary diagnoses (mood, anxiety, psychotic, and neurodevelopmental disorders) will therefore be considered as covariates. Intake of psychotropic medications are respectively frequent. The literature shows reciprocal interactions between psychotropic medications and gut microbiota (115), and metabolic patterns in response to treatment (116, 117). Type of medication class intake (antipsychotics, antidepressants, antianxiety drugs, anticonvulsants/mood stabilisers, opioid substitution) will be considered as covariates. Length and consumption patterns from SUD history will also enter covariate analysis (with regard to substances – primarily consumed substances vs. polytoxicomania). These covariates will be entered in Multi-omics factor analysis (MOFA) in direct joint modeling (118) to ensure that observed microbiome–metabolome–psychological associations are not driven by covariate effects. For the microbiome domain, intervention vs. control group comparisons regarding microbial abundance and composition will be performed with Analysis of Compositions of Microbiomes with Bias Correction (ANCOM-BC), a package which allows for continuous and categorical covariate adjustment within a linear regression framework, and was designed to analyze longitudinal data (119).

Metabolome and microbiome studies involve the examination and statistical testing of a multitude of variables. Thus, controlling for multiple testing is a must; False Discovery Rate corrections will be based on statistical recommendations promoting flexible algorithms within the R package FDRestimation (120).

#### 4.7.4 Statistical analyses of psychological variables

The statistical analyses will be performed in SPSS version 29 (IBM) for general procedures, while specific regression models are handled in R (R core team (121)). For example, t-tests will be carried out for independent and paired comparisons of means between and within groups at different times, respectively, assuming that the continuous data are normally distributed. Chi-square and Fisher’s exact tests, the latter in cases of smaller sample sizes, will be conducted in order to test the associations between categorical variables. The Friedman test shall be used in analyzing non-parametric repeated measures. The main effects of the intervention, time effects, and group x time interaction will be tested using a 2 × 3 two-way repeated measures ANOVA design (group x time), using the Greenhouse-Geisser correction in cases when the sphericity assumption will be violated. The level of significance will be α = 0.05, two-tailed, using Bonferroni adjustment for multiple comparisons. In addition, a repeated measures proportional odds logistic regression model using a generalized estimating equation (GEE) will be applied, assessing the intervention’s effect on CPD treated as an ordinal variable. The GEE model, computed in R using the `repolr` package (122), will contain robust variance estimators that take into account within-subject correlations (**Table 2**).

**Table 2:** Summary of statistical methods and outcomes.

Outcome variable	Domain	Statistical method	Software
Self-efficacy, craving, psychiatric scores	Psychobehavioural	Repeated measures ANOVA, t-tests, Friedman test	SPSS
Cigarettes per day (ordinal)	Psychobehavioural	GEE (Proportional odds model)	R (repolr)
Bacterial diversity indices	Microbiome	Kruskal-Wallis, ANOSIM, db-RDA	R, QIIME
Taxonomic composition	Microbiome	MaAsLin2, ANCOM, LEfSe	QIIME2, R
Metabolite concentrations	Metabolic	PCA, O-PLS-DA, correlation analyses	NMR tools, R
Cross-domain associations	Combined (exploratory)	Correlation and regression	R

#### 4.7.5 Microbiome analyses

One gram of collected stool sample will be immediately stored in a -80 °C-freezer. Sequence analysis will be done according to the supplier’s recommendations. The workflow for microbiome analysis with Illumina MiSeq has already been described in detail in Klymiuk et al. (123). The Magna Pure LC DNA III Isolation Kit (Bacteria) (Roche, Mannheim, Germany) will be used to extract DNA according to manufacturer’s instructions. The hypervariable V3–V4 regions of the bacterial 16S rRNA gene will be amplified with Polymerase-chain-reaction (PCR) from total fecal DNA using the target-specific primers. 2µl of total DNA will be used in a 25µl PCR reaction in triplicates with the FastStart High Fidelity PCR system (Sigma, Germany). Cycling condition of initial denaturations at 95 °C for 3 minutes, followed by 30 cycles of 95 °C for 45 sec, 55 °C for 45 sec, 72 °C for one minute and a final elongation step at 72 °C for seven minutes. The resulting amplification products will be visualized on a 1.5% agarose gel and pooled, indexed and purified as described in Klymiuk et al. (123). The final library will be sequenced at ZMF Core Facility Molecular Biology in Graz, Austria, using an Illumina MiSeq desktop sequencer with v3 chemistry and 600 cycles (2x300). Unmapped bam files will be used as input for bioinformatics. To analyze the microbial community structure and taxonomic diversity, the obtained raw reads will be processed using Quantitative Insights Into Microbial Ecology (QIIME) scripts. During processing in silico, the FASTQ files will be paired, demultiplexed, dereplicated, and quality controlled. Further processing will include the use of the DADA2 algorithm (124) for sequence inference and chimera removal. Low-abundance features will be removed below a threshold of 20 total counts and presence in less than 5 samples. Sequences will be aligned against the SILVA 132 database, using the Scikit-learn classifier for taxonomic identification. The MAFFT software will be employed for *de novo* multiple alignment; the resulting alignment will be further treated with FastTree for phylogenetic tree construction (125). The Pielou’s Evenness Index considers species evenness and is similar to the Shannon-Wiener diversity model. Besides, Faith’s Phylogenetic Diversity (PD) accounts for phylogenetic differences among species. Numbers of detected species will be used for approximating richness, and Shannon Index for overall diversity estimates. Analyses of alpha diversity will be done in R (4.2) and RStudio. The pairwise Kruskal-Wallis test will be used to make statistical comparisons between the smoking cessation intervention and control groups.

For beta diversity, db-RDA will be performed on Bray-Curtis, Jaccard, and UniFrac distance matrices (weighted and unweighted) to compare the structural similarities between groups’ microbiomes. Beta diversity indices will be visualized with PCoA plots using Bray-Curtis and Jaccard dissimilarities. The Firmicutes/Bacteroidetes (F/B) ratio will be calculated from the rarefied feature table. Using this, MaAsLin2 will be used to detect differentially abundant taxa and further test associations with variables of interest such as cigarettes per day or metabolic markers (126). Identifications of microbial biomarkers will then be done applying LeFSe both in longitudinal and cross-sectional comparisons. This will be furthered to detect significant differences between groups in the microbial taxonomic abundance using the Analysis of Composition of Microbiomes’ ANCOM-BC implemented within QIIME2 (119).

#### 4.7.6 Metabolome analyses

##### 4.7.6.1 Nuclear magnetic resonance (NMR) pipeline

To quantify metabolites in serum and stool of the participants, the untargeted metabolomics analysis will be performed with state-of-the-art NMR (nuclear magnetic resonance). Nuclear magnetic resnonance (NMR) spectroscopy was selected over other metabolomics pipelines such as mass-spectrometry (MS-) based approaches. Liquid chromatography (LC-MS) and gas chromatography (GC-MS) allow for comprehensive, untargeted metabolome profiling and analyses of specific metabolic pathways (127). However, the strength of NMS spectroscopy is that it provides a robust and highly reproducible platform for metabolomics, offering inherent quantitative accuracy regarding analytical stability, quantitative reliability, and data consistency (128, 129). NMR is therefore well suited for a longitudinal study design, allowing for excellent comparability of groups and timepoints. Metabolome analysis will focus on gut bacteria metabolites such as SCFA (e.g., butyrate), processed nutrients like polyphenols, folate, tryptophan and kynurenine metabolites, and metabolites of biochemical energy-pathways. Furthermore, choline and folate levels, lipids, and metabolites of inflammatory pathways and oxidative stress will be analysed.

##### 4.7.6.2 NMR analysis of stool metabolites

For NMR analysis of stool metabolites, to account for inter-individual variability in fecal water content, an aliquot of each stool sample will be lyophilized, and metabolite concentrations and microbial abundances will be normalized to fecal dry weight. Samples will then be mixed with 500 with 500 μl NMR buffer in D2O and transferred into 5-mm NMR tubes. NMR measurements will be performed on an AVANCE™ Neo Bruker Ultrashield 600 MHz spectrometer equipped with a TXI probe head at 310 K. The 1D CPMG (Carr-Purcell_Meiboom_Gill) pulse sequence with water suppression using pre-saturation will be used for 1H 1D NMR experiments. Bruker Topspin version 4.0.2 will be used for NMR data acquisition. The spectra for all samples will be automatically processed (exponential line broadening of 0.3 Hz), phased and referenced using TSP at 0.0 ppm using the Bruker Topspin 4.0.2 software (Bruker GmbH, Rheinstetten, Germany). Spectra pre-processing and data analysis will be carried out using the state-of-the-art data analysis pipeline (group of Prof. Jeremy Nicholson at Imperials College London) using Matlab^®^ scripts and MetaboAnalyst 4.0 (130). NMR data will be processed with Matlab^®^ vR2014a (Mathworks, Natick, Massachusetts, USA), and regions around the water, TSP, and remaining methanol signals will be excluded, and probabilistic quotient normalization will be applied to correct for sample metabolite dilution (131).

##### 4.7.6.3 NMR analysis of plasma metabolites

Blood plasma low molecular weight metabolites and lipoproteins will be also be analyzed on a Bruker 600 MHz Avance Neo NMR spectrometer. Plasma samples will be thawed, and 330 μl of each sample will be mixed with 330 μl of Bruker plasma buffer (Bruker, Rheinstetten, Germany). Following gentle mixing, 600 μl of the samples will be transferred into 5mm glass tubes and placed into a SampleJet rack (Bruker, Rheinstetten, Germany). Proton spectra will be ascertained at a temperature of 310 K using a standard nuclear Overhauser effect spectroscopy (NOESY) pulse sequence (Bruker: noesygppr1d), a Carr–Purcell Meiboom–Gill (CPMG) pulse sequence with pre-saturation during the relaxation delay (Bruker: cpmgpr1d) to achieve water suppression, and a fast scan 2D J-resolved (JRES) pulse sequence (Bruker: jresgpprqf). Data analyses will be carried out using Bruker IVDr Plasma (B.I.) module of the analysis software (Topspin version 4.1). Univariate statistical analysis of the concentration values for identifying lipoproteins and metabolites of interest differing between groups will again be carried out using MetaboAnalyst and the R package rstatix.

#### 4.7.7 Methods of preventing bias

In the present study, several measures are taken to avoid bias. First, randomization is performed to ensure that participants are randomly assigned to the different groups. Furthermore, an experimental design with a control group that receives the usual treatment, allows a comparison with the intervention group. Additionally, all subjects are housed in the same facility where they receive the same treatments, meals, and – for the intervention group – the same smoking cessation intervention. These conditions ensure a high degree of uniformity and standardization, minimizing potential bias. Dietary factors, and confounders such as medication will be considered cautiously and explicitly (see 4.7.2 and 4.7.3).

## 5 Collaboration

The study is a joined project between the Outpatient Counselling and Care Centre “Grüner Kreis “Graz, the Medical University Graz and the Sigmund Freud Private University Vienna.

The task of the Care Centre “Grüner Kreis” in Graz is to provide patients for the study and transport them to the clinical facility and back.

The task of the Medical University Graz, represented by Prof. Sandra Holasek, is to conduct the clinical study, including collection of samples and sample analysis.

The tasks of the Sigmund Freud Private University, represented by the Principal Investigator Prof. Human-Friedrich Unterrainer, are the project organization and management, data analysis and interpretation.

## 6 Work plan and timeline

**Table 3** displays the planned work timeline of the project.

**Table 3:** Work plan of the project.

Work packages	Year 1				Year 2				Year 3			
	1-3	4-6	7-9	10-12	1-3	4-6	7-9	10-12	1-3	4-6	7-9	10-12
WP 1 Project management												
WP 2 Patient enrolment & clinical trial												
WP 3 Biomarker analysis												
WP 4 Statistical analysis & Bioinformatics												
WP 5 Publication & Dissemination												

green cells: engagement in respective activity

## 7 Ethical aspects

The study will be conducted in accordance with the ethical guidelines of the Declaration of Helsinki as well as national and international regulations. It will be conducted according to the protocol and the requirements of the concerned regulatory authorities. Participants will be informed in detail about the objectives, methods, potential benefits and risks of the study. Participation is voluntary and based on a written declaration of consent. Participants’ personal data will be treated in strict confidence and used exclusively in anonymized form for data analysis. The risks related to the burden on participants include both psychological stress and physical discomfort that may be caused by nicotine withdrawal. However, given the participants’ willingness to quit smoking, they are consciously choosing a cessation program, which means the potential knowledge gain outweighs the temporary burdens of nicotine withdrawal. Furthermore, there will always be a clinical psychologist and a trained support system at ‘Grüner Kreis’ where the patients can receive professional guidance and support. As already mentioned in 4.6, providing a stool sample carries no risk. However, blood sampling can result in a bruise (hematoma), and depending on the condition of the veins, it can be more or less painful. This will be performed by an experienced study physician in accordance with WHO guidelines, who will also offer immediate support if necessary. The trial will be registered at the US-governmental repository www.clinicaltrials.gov before the enrolment of the first participant.

## 8 Sex-specific and gender-related issues

In this study, sex, gender, age, and ethnic background do not influence the selection of the participants. It is aimed for a representative balance at all levels. However, it is possible that the number of male participants predominates, as they have been shown to have a higher prevalence of SUDs (132). Also, the preliminary data from the pilot study show a male predominance among study patients. Sex can evidently influence acute and long-term effects as well as side effects and treatment outcomes in SUDs, which is why sex as a variable will be incorporated in the statistical analysis plan (132, 133). Equal opportunities for all patients interested in participating in a smoking cessation intervention will be provided.
